# Supplementary material for: Dose-dependent effects of rumen-protected choline on hepatic metabolism during induction of fatty liver in dry pregnant dairy cows
Source: PLoS One. 2023 Oct 5;18(10):e0290562. doi: 10.1371/journal.pone.0290562 (PMC10553221; doi:10.1371/journal.pone.0290562)
Supplement: S1 Table — (DOCX) [file pone.0290562.s001.docx]

| **Supplemental Table S1. Dietary ingredients and nutrient content of diets fed during the ad libitum and feed restriction periods** | | |
| --- | --- | --- |
|  | **Diet^1^** | |
| **Ingredient, % dry matter basis** | **Ad libitum** | **Feed restriction** |
| Corn silage | 33.70 | 38.40 |
| Triticale silage | 20.10 | 22.90 |
| Bermudagrass hay | 6.67 | 7.52 |
| Wheat straw | 13.60 | 15.50 |
| Citrus pulp, dried | 7.60 | -- |
| Soybean meal | 12.90 | -- |
| Prepartum mineral^2^ | 4.20 | 11.20 |
| Corn grain, ground | 0.81 | 2.27 |
| Molasses | 0.27 | 0.76 |
| Salt | 0.15 | 0.60 |
| Rumen-protected methionine^3^ | --- | 0.85 |
| Nutrient content,^4^ dry matter basis |  |  |
| Crude protein, % | 14.8 | 10.1 |
| Neutral detergent fiber, % | 42.9 | 51.6 |
| Starch, % | 12.5 | 16.2 |
| Crude fat, % | 2.72 | 2.95 |
| Net energy for lactation,^5^ |  |  |
| Mcal/kg | 1.61 | 1.48 |
| Mcal/day | 14.7 | 4.90 |
| Metabolizable^5^ |  |  |
| Protein, g/day | 925 | 235 |
| Methionine, g/day | 20 | 20 |
| Methionine, % of metabolizable protein | 2.16 | 8.51 |

^1^ Ad libitum = diet fed to cows for ad libitum intake on days 1 to 5; feed restriction = diet fed to cows during the feed restriction period, on days 6 to 14 of the experiment.

^2^ Contained (dry matter basis) 62.7% corn gluten feed, 15.7% magnesium sulfate × 7 H_2_O, 8.9% calcium carbonate, 6.7% magnesium oxide, 2.2% sodium chloride, 2.0% ClariFly Livestock Premix 0.67% (Central Garden & Pet Co, Schaumburg, IL), 0.67% vitamin E 500,000 kIU/kg, 0.45% Sel-Plex 2000 (Alltech Biotechnology, Nicholasville, KY), 0.38% Rumensin 90 (Elanco Animal Health, Greenfield, IN), 0.27% IntelliBond Vital 4 (Micronutrients USA LLC, Indianapolis, IN), 0.036% vitamin A concentrate 650,000 kIU/kg, 0.013% vitamin D concentrate 500,000 kIU/kg, and 0.002% ethylenediamine dihydroiodide 80%. Each kilogram contained 13.5% CP, 3.7% Ca, 0.9% P, 5.5% Mg, 0.9% K, 2.3% S, 1.0% Na, 1.6% Cl, 151 mg of Fe, 724 mg of Zn, 165 mg of Cu, 543 mg of Mn, 9 mg of Se, 4 mg of Co, 16 mg of I, 233 kIU of vitamin A, 67 kIU of vitamin D, 3,362 IU of vitamin E, 748 mg of monensin, and 139 mg of diflubenzuron.

^3^ Smartamine M, Adisseo NA (Alpharetta, GA) containing 60% metabolizable methionine.

^4^ Based on chemical analyses of feeds [7].

^5^ Estimated using NASEM [19] based on the chemical analyses of individual ingredients and the observed mean dry matter intakes during the ad libitum and feed restriction periods.
