# Supplementary material for: Dose-dependent effects of rumen-protected choline on hepatic metabolism during induction of fatty liver in dry pregnant dairy cows
Source: PLoS One. 2023 Oct 5;18(10):e0290562. doi: 10.1371/journal.pone.0290562 (PMC10553221; doi:10.1371/journal.pone.0290562)
Supplement: S2 Table — (DOCX) [file pone.0290562.s002.docx]

| **Supplemental Table S2. List of transcripts and primers investigated by real time PCR analysis of hepatic tissue segregated according to function** | | | |
| --- | --- | --- | --- |
| **Category gene^1^** | **Primer** | **Accession number** | **Efficiency, %** |
| Housekeeping |  |  |  |
| *ACTB* | F: 5’-GCCAACCGTGAGAAGATGAC-3’  R: 5’-CCTGGATGGCCACGTACA-3’ | NM_173979 | 112.2 |
| *RPL12* | F: 5’-ATGCGGCATCGGTCTCTA-3’  R: 5’-AACATTGCAGCCCACAGAC-3’ | NM_205797 | 107.2 |
| *RPS9* | F: 5’-GCCTCGACCAAGAGCTGAA-3’  R: 5’-GAATTTGACCCTCCAGACCTCA-3’ | NM_001101152 | 113.3 |
| Choline metabolism |  |  |  |
| *BHMT* | F: 5’-TGCTGTGGAACACCCAGAA-3’  R: 5’-AGGTGAAGGTCTGCATGACA-3’ | NM_001011679 | 107.2 |
| *CBS* | F: 5’-TGGAAGGGATCGGCTATGAC-3’  R: 5’-CTCCTCATCGTTGCTCTTGAAC-3’ | NM_001102000 | 118.8 |
| *CEPT1* | F: 5’-AGTCTTCTACTGCCCTACAGCTA-3’  R: 5’-AAATGAAAAGGCCGCATGCA-3’ | NM_001193130 | 115.4 |
| *CHDH* | F: 5’-GTGGGTGTGGAGTACCTCAAA-3’  R: 5’-GATGGCACCTCCACTCAGAA-3’ | NM_001205564 | N/A |
| *CHKA* | F: 5’-GGTTTACCGGGGAGTCCAAA-3’  R: 5’-GACCTCAGGTTTTCTAGCTCCAA-3’ | FLDM_052176 | 111.2 |
| *CHPT1* | F: 5’-GGAGCAACAATGTGGGACTA-3’  R: 5’-TGCTCCACCTACTACTCCAA-3’ | NM_001075507 | 111.7 |
| *GAMT* | F: 5’-GGGGAGAGCTAATGAAGACCAA-3’  R: 5’-TGACCTGTGTGCGGATGTTA-3’ | NM_001038544 | 112.2 |
| *GNMT* | F: 5’-TTTTGCCCACTTGCCAGAC-3’  R: 5’-CATGCTTGCGATGTTCCTCA-3’ | NM_001206116 | 114.9 |
| *MAT1A* | F: 5’-GTGCTGGATGCCCATCTCAA-3’  R: 5’-GAAGCACCATGCCCGTCTTA-3’ | NM_001046497 | 114.9 |
| *MTHFR* | F: 5’-CCAGGGTGCTGTCAATCTCA-3’  R: 5’-TGCCAGGTCACGTCTACAAA-3’ | NM_001011685 | 118.3 |
| *MTR* | F: 5’-TGTGGTACGACACCAGATCA-3’  R: 5’-GAAAACAGTGGCAGGTGGAA-3’ | NM_001030298 | 107.2 |
| *PCYT1A* | F: 5’-AACCTCCAGAGGGGCTACA-3’  R: 5’-CAACTCGCTCCTGTAAGTGGTA-3’ | NM_001105052 | 107.7 |
| *PCYT1B* | F: 5’-CGAGGGAATTCATTGGTAACTTCC-3’  R: 5’-GTTGCTCCTCTCCTGGAACA-3’ | NM_001193051 | N/A |
| *PEMT* | F: 5’-CTGGTGGCGCTCATCTACA-3’  R: 5’-CTGGGAGGCTTTCTGCTGATA-3’ | NM_182989 | 112.7 |
| *PLD1* | F: 5’-GCCTTTATCCGAATTCCCATTCC-3’  R: 5’-TCTCTCGAGGCTCTTCTTTGAC-3’ | NM_001102001 | 99.7 |
| Insulin biosynthesis |  |  |  |
| *PCSK1* | F: 5’-GCAGAGGAGACCTTCATGTCA-3’  R: 5’-GGTGTCCCGCTCTCTTTCA-3’ | NM_174412 | N/A |
| Fibroblast growth factor |  |  |  |
| *FGF21* | F: 5’-GGCATCATCCGTGTAGAGGT-3’  R: 5’-TTCAAGCACTTGGGACTGTG-3’ | GEP00085659 | 108.7 |
| Gluconeogenesis |  |  |  |
| *G6PC* | F: 5’-GGCATCAAACTCCTCTGGGTA-3’  R: 5’-AGTATGGGCGCTGTCCAAA-3’ | NM_001076124 | 108.7 |
| *PCK1* | F: 5’-GAGGAGGAGGGTGTGATCAA-3’  R: 5’-TTCAATTCTGGCCACATCCC-3’ | NM_174737 | 115.4 |
| *PCK2* | F: 5’-AGCAGGGACTCATCCGAAAA-3’  R: 5’-TCTCTACTCGTGCCACATCC-3’ | NM_001205594 | 109.2 |
| *SLC2A1* | F: 5’-GCTGTGCAGTGCTCATGAC-3’  R: 5’-ATGGCCACAATGCTCAGGTA-3’ | NM_174602 | N/A |
| *SLC2A2* | F: 5’-GGTGTGATCAATGCACCTCA-3’  R: 5’-TCGGTCATCCAGTGAAACAC-3’ | NM_001103222 | 110.2 |
| Fatty acids (FA) trafficking |  |  |  |
| *CD36* | F: 5’-GCATTGAAGAATCTGAAGCACAAC-3’  R: 5’-GCCTTCTCATCACCAATGGTAC-3’ | NM_001278621 | 106.3 |
| *FABP1* | F: 5’-GGAGTTCATGACTGGGGAGAA-3’  R: 5’-TGATGCCCTTGAAAGTTGTCAC-3’ | NM_175817 | 112.7 |
| *SLC27A1* | F: 5’-TTCTGGGATGACTGCGTCAA-3’  R: 5’-CGGCTGCTTCAGCAGGTA-3’ | NM_001033625 | N/A |
| *SLC27A2* | F: 5’-TCCCTGTTGCACTGCTTTCA-3’  R: 5’-TTGGCAGCACCTCTTCAACA-3’ | NM_001192863 | 110.2 |
| *SLC27A5* | F: 5’-GCTTGTCCTTGGAGTCCTCA-3’  R: 5’-GAAGCCGGAGGCAGAGAA-3’ | NM_001103273 | N/A |
| Carnitine metabolism |  |  |  |
| *BBOX1* | F: 5’-TTTTCACAGCAGGCCAGAGAA-3’  R: 5’-TGGAACTCTGAGCCCCAGTA-3’ | NM_001101881 | 106.8 |
| *CRAT* | F: 5’-GTGTGATGCTGCCAAAGCA-3’  R: 5’-TCCAACACGCCCTCGATTAA-3’ | NM_001075587 | 116.6 |
| *CROT* | F: 5’-GCACTTGGCAGTCCTAAACA-3’  R: 5’-CGATGGAAGGGAATCTTGGTAC-3’ | NM_177494 | 106.8 |
| *SLC22A5* | F: 5’-GGGATCATTGCACCTTCAACC-3’  R: 5’-ACTGCTGCTTCTTGGAGCTTA-3’ | NM_001046502 | N/A |
| *TMLHE* | F: 5’-TGGCAGCAACATGAAGATCA-3’  R: 5’-TGGTCTCGAAGCCAGACATA-3’ | NM_001076064 | 108.7 |
| FA activation and oxidation |  |  |  |
| *ACADM* | F: 5’-AGCCTGGGAGCTTGGTTTAA-3’  R: 5’-TCAAAAGTTCCAAGTCCAAGACC-3’ | NM_001075235 | 108.2 |
| *ACSL1* | F: 5’-GGAAGAGCCAACAGACAGAA-3’  R: 5’-GTAGTTCCACTGGTGAAGCA-3’ | NM_001076085 | 107.7 |
| *CPT1A* | F: 5’-ATCCACGCCATCCTGCTTTA-3’  R: 5’-GGAATCGTAGACCCCAGAAGAA-3’ | NM_001304989 | 107.2 |
| *PPARA* | F: 5’-GACAAAGCCTCTGGCTACCA-3’  R: 5’-CTTCAGCCGAATCGTTCTCCTA-3’ | NM_001034036 | 107.7 |
| *PPARG* | F: 5’-CTTGCTGTGGGGATGTCTCA-3’  R: 5’-ATCTCCGCTAACAGCTTCTCC-3’ | NM_181024 | N/A |
| *SLC25A20* | F: 5’-GGGGAAGAAACTGCAACAGAAA-3’  R: 5’-GACAACATCCCAGCTGCAAA-3’ | NM_001077936 | 110.7 |
| Synthesis or re-esterification of FA | |  |  |
| *ACACA* | F: 5’-GCTAACTCAACTCAGCAAGACC-3’  R: 5’-GGATGGCAAATGGGAAGCAA-3’ | NM_174224 | 103.1 |
| *ACLY* | F: 5’-ACCAGAAGGGAGTGACCATCA-3’  R: 5’-GCCGGTGTTCCCGATCTTAA-3’ | NM_001037457 | N/A |
| *FASN* | F: 5’-GCCCCTACTTCCAAGGTATCC-3’  R: 5’-GTCACCCAGTTGTCCTTCCA-3’ | NM_001012669 | N/A |
| *DGAT2* | F: 5’-TGGCTGGTGTTTGACTGGAA-3’  R: 5’-TCGAAAGTAGCGCCACACA-3’ | NM_205793 | 98.4 |
| Phospholipid metabolism |  |  |  |
| *AGPAT2* | F: 5’-ACTCCAGCTTCTCCTCCTTCTA-3’  R: 5’-GATGGCATCTAGCACCTCCA-3’ | NM_001080264 | 108.7 |
| *AGPAT3* | F: 5’-AGCAGCTCAGTGGCTTCATA-3’  R: 5’-AGGAAACACACCCTTCTGGTTA-3’ | NM_001038046 | 109.7 |
| *PLA2G1B* | F: 5’-TCCCTACACCAACAACTACTCC-3’  R: 5’-TTGCAGATGAAGGCCTCACA-3’ | NM_174646 | N/A |
| *PTTDS1* | F: 5’-GGTCTCTGCTGGACAATCAGTA-3’  R: 5’-GCACTCGGCAAAATTGGGTA-3’ | NM_001046040 | 103.5 |
| *SGMS1* | F: 5’-GATCCTTGTGGGACTCTGGTTAA-3’  R: 5’-GCGTGCCAACTATGCAGAAA-3’ | NM_001206135 | N/A |
| *SMPD1* | F: 5’-CACCACCTACATCGGCCTTAA-3’  R: 5’-ACGTGAGAGCTCCCAGAGTA-3’ | NM_001075187 | 112.2 |
| Lipoprotein synthesis and assembly | |  |  |
| *APOB100* | F: 5’-ACAGTGTCAACAAGGCTTTGTAC-3’  R: 5’-TAGCCAAAGTGGTCCACCAA-3’ | FLDM_067263 | 111.7 |
| *APOE* | F: 5’-GCTCAACACCCAGGTCATTCA-3’  R: 5’-CCCTCCAGCTCCTCCTTGTA-3’ | NM_173991 | 108.2 |
| *HDLBP* | F: 5’-GCCTTGAGATCATGCAGAGAAC-3’  R: 5’-GTCCAGCTTCCCTGAGACC-3’ | FLDM_002175 | 110.7 |
| *MTTP* | F: 5’-TGCTCTCCATTGTCCAAGAC-3’  R: 5’-GCGACCATTTCCTTCAGAAC-3’ | NM_001101834 | 107.2 |
| *PCTP* | F: 5’-GCAAGTGAAGTACCCTTTTCCC-3’  R: 5’-TGGACCTTCTGCCCTTCAAA-3’ | NM_174835 | 107.2 |
| *VLDLR* | F: 5’-ATGGCAGTGACGAGAAGAAC-3’  R: 5’-CACTGGCCATTGTTGCATAC-3’ | NM_174489 | N/A |
| Cholesterol metabolism |  |  |  |
| *ABCA1* | F: 5’-GGTGTCTTGGCAGTGTTCAA-3’  R: 5’-GTTCGACCCTGCTATTCGTAC-3’ | NM_001024693 | 105.8 |
| *ABCG1* | F: 5’-GGGAAGTCCACACTCATGAACA-3’  R: 5’-GGAGCCCGTTGATGAGGAC-3’ | NM_001205528 | 111.2 |
| *CYP7A1* | F: 5’-TCAGACACAGCTGGACAACA-3’  R: 5’-TGTTGAGGGAAGCACTGGAA-3’ | NM_001205677 | 103.1 |
| *FDPS* | F: 5’-GAGATGCCATTACCCGACTCA-3’  R: 5’-ACCGTCAAACCCCGATTGTA-3’ | NM_177497 | 112.7 |
| *MVK* | F: 5’-GCTGGCCTTCCTCTACTTGTA-3’  R: 5’-GGCAGCTCCGACCATACA-3’ | NM_001015528 | 109.7 |
| Cell signaling |  |  |  |
| *NR1H3* | F: 5’-GCCTTGCTCATTGCCATCA-3’  R: 5’-TGTGTGTTGCAGCCTCTCTA-3’ | NM_001014861 | 109.7 |
| De novo hepatic lipogenesis |  |  |  |
| *SREBF1* | F: 5’-CGCTACCGCTCTTCCATCAA-3’  R: 5’-TGCGCAAGACGGCAGATTTA-3’ | NM_001113302 | 104.4 |
| *SREBF2* | F: 5’-CCCGTCATCTACCAGAATGCA-3’  R: 5’-ACCTGGGAGGAGGTCACAA-3’ | NM_001205600 | N/A |
| Ketogenesis |  |  |  |
| *ACAT1* | F: 5’-GAGCTGTTTCTCTTGGACATCC-3’  R: 5’-GCTTCAGTGCATGAGCCAAA-3’ | NM_001046075 | 109.2 |
| *HMGCL* | F: 5’-TCCACTGCCATGACACCTAC-3’  R: 5’-AAGAGTCCATGACACTCACTCC-3’ | NM_001075132 | 116.3 |
| *HMGCR* | F: 5’-TGATGCCATGGGGATGAACA-3’  R: 5’-CTGCATTTCGGGGAAATACTCC-3’ | NM_001105613 | 110.7 |
| *HMGCS1* | F: 5’-TGGAGCCGTTGCTATGCTA-3’  R: 5’-GTTGCATATGTGTCCCACGAA-3’ | NM_001206578 | 102.7 |
| Cytokines |  |  |  |
| *IL1B* | F: 5’-TGTGTGCTGAAGGCTCTCC-3’  R: 5’-CCTTGCACAAAGCTCATGCA-3’ | NM_174093 | 116.0 |
| *IL10* | F: 5’-CCCTGCGAAAACAAGAGCAA-3’  R: 5’-CTCACTCATGGCTTTGTAGACAC-3’ | NM_174088 | 130.3 |
| *MGST3* | F: 5’-AGGCCCGCAAGAAGTACAA-3’  R: 5’-GCTCGCTGAATGCAGTTGAA-3’ | NM_001035046 | 106.8 |
| *TNFA* | F: 5’-CAAGTAACAAGCCGGTAGCC-3’  R: 5’-GGCATTGGCATACGAGTCC-3’ | NM_173966 | N/A |
| Acute phase proteins |  |  |  |
| *CRP* | F: 5’-GAGGCTGTTGTGGTGTTTCC-3’  R: 5’-AACACAAAGGCCTTCTTATGCA-3’ | NM_001144097 | 108.7 |
| *HP* | F: 5’-CTTCTCGCGGTGGAAACC-3’  R: 5’-CCGAGTACTCCACATGGCTA-3’ | NM_001040470 | 112.7 |
| *SAA3* | F: 5’-ACATTCCTCAAGGAAGCTGGT-3’  R: 5’-ACCCCTGTAGTTGGCTTCTTT-3’ | NM_181016 | 113.3 |
| Oxidative stress |  |  |  |
| *HMOX2* | F: 5’-CCAACAAGGCCTTTGAGTTCA-3’  R: 5’-GGGTTTCTTTGGCCAGCAA-3’ | NM_001035087 | 114.9 |
| Synthesis of antioxidants |  |  |  |
| *CAT* | F: 5’-TGAATATGGCTCCCGCATCC-3’  R: 5’-CATGCTGCACATAGGTGTGAAC-3’ | NM_001035386 | 108.7 |
| *GPX3* | F: 5’-AGCAGTACGCTGGCAAATAC-3’  R: 5’-CTCTTCCTGCAGTGCATTCA-3’ | NM_174077 | 108.7 |
| *MT1A* | F: 5’-TCCGACCAGTGGATCTGCTT-3’  R: 5’-AGGAGCAGTTCGGGTCCATT-3’ | NM_001040492 | 112.2 |
| *MT1E* | F: 5’-CCAAATGGACCCCAATTGCT-3’  R: 5’-GGCATCTGCAGGCCTTG-3’ | NM_001078134 | 114.9 |
| *MT2A* | F: 5’-CGGCTCCTGCAAATGCAAA-3’  R: 5’-CGAAGCCCCTTTGCAGAC-3’ | NM_001075140 | 113.3 |
| *NQO1* | F: 5’-CTCATAGGGGAGTTCGCTTACAA-3’  R: 5’-ACCAGTGGTGATGGAAAGCA-3’ | NM_001034535 | 110.2 |
| *SOD1* | F: 5’-CGGTGTTGCCATCGTGGATA-3’  R: 5’-TCCACCTCTGCCCAAGTCA-3’ | NM_174615 | 110.7 |
| Pathogen recognition receptor |  |  |  |
| *TLR4* | F: 5’-AGGCAGCCATAACTTCTCCA-3’  R: 5’-AGGGTTTCCCGTCAGTATCAA-3’ | NM_174198 | N/A |
| Lipid peroxidation |  |  |  |
| *ALDH7A1* | F: 5’-AGCTCCTACAACTTCCCTCA-3’  R: 5’-CAGCTTGTTGTCCTCCAGAA-3’ | NM_001045969 | 110.7 |
| *ALDH9A1* | F: 5’-TTTCAGATCGCCTGCTGGAA-3’  R: 5’-GGCGTGAAGGGAGAAGGTTTA-3’ | NM_001046423 | 109.2 |
| Glycerol phosphate pathway |  |  |  |
| *GDE1* | F: 5’-GGAAGCTTAATCCTGCAGCAAA-3’  R: 5’-ACTCTGCAACGGCTTCTCTTA-3’ | NM_001034686 | 120.6 |
| *GDPD1* | F: 5’-GGGTAACGCCAGCTATGAAA-3’  R: 5’-GAGCAGCACACGTTGTAAAC-3’ | NM_001076400 | N/A |
| *GDPD2* | F: 5’-TACAGCTGCCACTGGAAGAA-3’  R: 5’-TGAGGAAGAGCAGGCCAAA-3’ | NM_001034471 | N/A |
| *GPAM* | F: 5’-AAGAGGCCCTTTGTGGGAA-3’  R: 5’-ACGGGATACTGGGGTTGAAA-3’ | NM_001012282 | 114.9 |
| *GPD2* | F: 5’-CGTCCAGGACTATGGACTTGAA-3’  R: 5’-TGGCCACCTCAAAAGCCTTA-3’ | NM_001100296 | 100.5 |
| Urea cycle |  |  |  |
| *ASS1* | F: 5’-CGGTTTGAGCTCACCTGCTA-3’  R: 5’-TATAGAACTCGGGCATCCTCCA-3’ | NM_173892 | 112.7 |
| *OTC* | F: 5’-CCAGAGGCCGAAAACAGAAA-3’  R: 5’-TTCTGGAGCTGAGGCGAATA-3’ | NM_177487 | 106.8 |
| Glucuronidation reaction |  |  |  |
| *UGT1A1* | F: 5’-GGATCAACTGCGCTAGCAAA-3’  R: 5’-AACCACAATTCCGTGTTCTCC-3’ | NM_001105636 | 102.2 |
| Endoplasmic reticulum stress |  |  |  |
| *XBP1* | F: 5’-TGACTGAAGAGGAAGCAGAG-3’  R: 5’-CAATGCCATCAGAGTCCATG-3’ | GEP00085658 | 97.6 |

^1^ *ABCA1* = ATP binding cassette subfamily A member 1; *ABCG1* = ATP binding cassette subfamily G member 1; *ACACA* = acetyl-CoA carboxylase alpha; *ACADM* = acyl-CoA dehydrogenase medium chain; *ACAT1* = acetyl-CoA acetyltransferase 1; *ACLY* = ATP citrate lyase; *ACSL1* = acyl-CoA synthetase long chain family member 1; *ACTB* = actin beta; *AGPAT2* = 1-acylglycerol-3-phosphate O-acyltransferase 2; *AGPAT3* = 1-acylglycerol-3-phosphate O-acyltransferase 3; *ALDH7A1* = aldehyde dehydrogenase 7 family member A1; *ALDH9A1* = aldehyde dehydrogenase 9 family member A1; *APOB100* = apolipoprotein B100; *APOE* = apolipoprotein E; *ASS1* = argininosuccinate synthase 1; *BBOX1* = gamma-butyrobetaine hydroxylase 1; *BHMT* = betaine--homocysteine S-methyltransferase; *CAT* = catalase; *CBS* = cystathionine-beta-synthase; *CD36* = cluster of differentiation 36; *CEPT1* = choline/ethanolamine phosphotransferase 1; *CHDH* = choline dehydrogenase; *CHKA* = choline kinase alpha; *CHPT1* = choline phosphotransferase 1; *CPT1A* = carnitine palmitoyltransferase 1A; *CRAT* = carnitine O-acetyltransferase; *CROT* = carnitine O-octanoyltransferase; *CRP* = c-reactive protein; *CYP7A1* = cytochrome P450 family 7 subfamily A polypeptide 1; *DGAT2* = diacylglycerol O-acyltransferase 2; *FABP1* = fatty acid binding protein 1; *FASN* = fatty acid synthase; *FDPS* = farnesyl diphosphate synthase; *FGF21* = fibroblast growth factor 21; *G6PC* = glucose-6-phosphatase catalytic subunit 1; *GAMT* = guanidinoacetate N-methyltransferase; *GDE1* = glycerophosphodiester phosphodiesterase 1; *GDPD1* = glycerophosphodiester phosphodiesterase domain containing 1; *GDPD2* = glycerophosphodiester phosphodiesterase domain containing 2; *GNMT* = glycine N-methyltransferase; *GPAM* = glycerol-3-phosphate acyltransferase, mitochondrial; *GPD2* = glycerol-3-phosphate dehydrogenase 2; *GPX3* = glutathione peroxidase 3; *HDLBP* = high density lipoprotein binding protein; *HMGCL* = 3-hydroxy-3-methylglutaryl-CoA lyase; *HMGCR* = 3-hydroxy-3-methylglutaryl-CoA reductase; *HMGCS2* = 3-hydroxy-3-methylglutaryl-CoA synthase 1; *HMOX2* = heme oxygenase 2; *HP* = haptoglobin; *IL1B* = interleukin 1 beta; *IL10* = interleukin 10; *MAT1A* = methionine adenosyltransferase 1A; *MGST3* = microsomal glutathione S-transferase 3; *MT1A* = metallothionein 1A; *MT1E* = metallothionein 1E; *MT2A* = metallothionein 2A; *MTHFR* = methylenetetrahydrofolate reductase; *MTR* = 5-methyltetrahydrofolate-homocysteine methyltransferase; *MTTP* = microsomal triglyceride transfer protein; *MVK* = mevalonate kinase; *NQO1* = NADPH quinone dehydrogenase 1; *NR1H3* = nuclear receptor subfamily 1 group H member 3; *OTC* = ornithine carbamoyltransferase; *PCK1* = phosphoenolpyruvate carboxykinase 1; *PCK2* = phosphoenolpyruvate carboxykinase 2; *PCSK1* = proprotein convertase subtilisin/kexin type 1; *PCTP* = phosphatidylcholine transfer protein; *PCYT1A* = phosphate cytidylyltransferase 1A; *PCYT1B* = phosphate cytidylyltransferase 1B; *PEMT* = phosphatidylethanolamine N-methyltransferase; *PLA2G1B* = phospholipase A2 group 1B; *PLD1* = phospholipase D1; *PPARA* = peroxisome proliferator activated receptor alpha; *PPARG* = peroxisome proliferator activated receptor gamma; *PTDSS1* = phosphatidylserine synthase 1; *RPL12* = ribosomal protein L12; *RPS9* = ribosomal protein S9; *SAA3* = serum amyloid A3; *SGMS1* = sphingomyelin synthase 1; *SLC22A5* = solute carrier family 22 member 5; *SLC25A20* = solute carrier family 25 member A20; *SLC27A1* = solute carrier family 27 member A1; *SLC27A2* = solute carrier family 27 member A2; *SLC27A5* = solute carrier family 27 member A5; *SLC2A1* = solute carrier family 2 member A1; *SLC2A2* = solute carrier family 2 member A2; *SMPD1* = sphingomyelin phosphodiesterase 1; *SOD1* = superoxide dismutase 1; *SREBF1* = sterol regulatory element binding transcription factor 1; *SREBF2* = sterol regulatory element binding transcription factor 2; *TLR4* = toll-like receptor 4; *TMLHE* = trimethyl lysine hydroxylase epsilon; *TNFA* = tumor necrosis factor alpha; *UGT1A1* = UDP glucuronosyltransferase 1 family, polypeptide A1; *VLDLR* = very-low density lipoprotein receptor; *XBP1* = splicing X-box binding protein 1.
