# Supplementary material for: Dose-dependent effects of rumen-protected choline on hepatic metabolism during induction of fatty liver in dry pregnant dairy cows
Source: PLoS One. 2023 Oct 5;18(10):e0290562. doi: 10.1371/journal.pone.0290562 (PMC10553221; doi:10.1371/journal.pone.0290562)
Supplement: S3 Table — (DOCX) [file pone.0290562.s003.docx]

| **Supplemental Table S3. Effect of amount of choline ion supplemented as rumen-protected choline on delta cycle threshold values of transcripts affected by treatment during the ad libitum period (LSM and SEM)** | | | | | | |
| --- | --- | --- | --- | --- | --- | --- |
|  | **Treatment^1^** | | | | |  |
| **Item^2^** | **0** | **6.5** | **12.9** | **19.4** | **25.8** | **SEM** |
| Choline metabolism |  |  |  |  |  |  |
| *BHMT* | 4.38 | 3.70 | 6.44 | 4.92 | 3.65 | 1.03 |
| *CEPT1* | 5.41 | 4.58 | 4.92 | 4.35 | 4.58 | 0.47 |
| *MTR* | 7.72 | 6.72 | 8.07 | 7.40 | 6.18 | 0.72 |
| Gluconeogenesis |  |  |  |  |  |  |
| *PCK2* | 2.99 | 1.79 | 1.59 | 0.34 | 0.09 | 0.53 |
| Activation of fatty acids |  |  |  |  |  |  |
| *ACSL1* | 1.58 | 2.06 | 2.02 | 2.04 | 2.44 | 0.37 |
| Oxidation of fatty acids |  |  |  |  |  |  |
| *PPARA* | 5.05 | 6.33 | 5.77 | 6.40 | 4.80 | 0.51 |
| Synthesis of phospholipids |  |  |  |  |  |  |
| *AGPAT2* | 6.09 | 5.98 | 5.34 | 4.84 | 4.78 | 0.61 |
| Lipoprotein synthesis and assembly |  |  |  |  |  |  |
| *APOE* | 7.36 | 5.95 | 5.94 | 5.54 | 5.08 | 0.93 |
| Cholesterol efflux |  |  |  |  |  |  |
| *ABCA1* | 5.70 | 5.30 | 5.75 | 4.30 | 4.08 | 0.63 |
| *FDPS* | 3.67 | 3.84 | 3.09 | 3.04 | 3.47 | 0.46 |
| De novo hepatic lipogenesis |  |  |  |  |  |  |
| *SREBF1* | 2.76 | 7.92 | 8.57 | 10.06 | 8.68 | 2.51 |
| Ketogenesis |  |  |  |  |  |  |
| *HMGCS1* | 1.24 | 0.85 | 1.28 | 1.05 | 0.66 | 0.29 |
| Acute phase response |  |  |  |  |  |  |
| *CRP* | -0.17 | -0.44 | 0.34 | 0.09 | -0.15 | 0.35 |
| Oxidative stress |  |  |  |  |  |  |
| *HMOX2* | 6.02 | 5.18 | 8.90 | 9.23 | 7.05 | 1.48 |
| Lipid peroxidation |  |  |  |  |  |  |
| *ALDH9A1* | 5.71 | 4.27 | 6.48 | 6.31 | 4.89 | 0.94 |
| Glycerol phosphate pathway |  |  |  |  |  |  |
| *GPD2* | 1.58 | 1.73 | 2.31 | 1.57 | 1.35 | 0.41 |
| Urea cycle |  |  |  |  |  |  |
| *OTC* | 4.80 | 2.97 | 5.50 | 5.24 | 4.50 | 1.08 |

^1^ Supplementation of 0, 6.45, 12.90, 19.35 or 25.80 g/d of choline ion as rumen-protected choline.

^2^ Cows were fed for ad libitum intake on days 1 to 5 and hepatic tissue collected in the morning of day 5, before imposing feed restriction.
