# Supplementary material for: Dose-dependent effects of rumen-protected choline on hepatic metabolism during induction of fatty liver in dry pregnant dairy cows
Source: PLoS One. 2023 Oct 5;18(10):e0290562. doi: 10.1371/journal.pone.0290562 (PMC10553221; doi:10.1371/journal.pone.0290562)
Supplement: S4 Table — (DOCX) [file pone.0290562.s004.docx]

| **Supplemental Table S4. Effect of amount of choline ion supplemented as rumen-protected choline on delta cycle threshold values of transcripts affected by treatment during the feed restriction period (LSM and SEM)** | | | | | | |
| --- | --- | --- | --- | --- | --- | --- |
|  | **Treatment^1^** | | | | |  |
| **Item^2^** | **0** | **6.5** | **12.9** | **19.4** | **25.8** | **SEM** |
| Choline metabolism |  |  |  |  |  |  |
| *BHMT* | 5.70 | 4.30 | 3.30 | 6.03 | 6.27 | 0.70 |
| *CBS* | 10.34 | 11.46 | 7.82 | 9.60 | 10.75 | 1.77 |
| *CHKA* | 5.58 | 4.09 | 3.32 | 2.58 | 2.22 | 0.59 |
| *MTR* | 5.97 | 7.56 | 5.44 | 5.67 | 6.94 | 1.00 |
| *PCYT1A* | 0.16 | -0.35 | -0.01 | -0.26 | -1.40 | 0.53 |
| *PLD1* | 1.06 | -0.22 | -0.12 | 0.14 | 0.06 | 0.54 |
| Growth factor |  |  |  |  |  |  |
| *FGF21* | 0.72 | 0.89 | 1.56 | 1.57 | 0.98 | 0.41 |
| Gluconeogenesis |  |  |  |  |  |  |
| *PCK1* | 3.64 | 3.33 | 2.65 | 2.71 | 3.75 | 0.57 |
| *PCK2* | 3.52 | 3.91 | 3.06 | 2.98 | 3.14 | 0.43 |
| Carnitine metabolism |  |  |  |  |  |  |
| *CRAT* | 5.76 | 6.63 | 4.90 | 4.60 | 7.27 | 1.29 |
| *TMLHE* | 6.47 | 7.95 | 6.21 | 6.59 | 7.50 | 0.82 |
| Activation of fatty acids |  |  |  |  |  |  |
| *ACSL1* | 2.38 | 2.78 | 2.17 | 1.70 | 2.08 | 0.43 |
| Oxidation of fatty acids |  |  |  |  |  |  |
| *CPT1A* | 4.91 | 6.73 | 2.87 | 3.86 | 4.55 | 1.47 |
| *PPARA* | 6.46 | 7.18 | 5.01 | 4.78 | 6.24 | 1.18 |
| Re-esterification of fatty acids |  |  |  |  |  |  |
| *DGAT2* | 0.61 | 1.95 | 3.04 | 4.26 | 5.24 | 0.81 |
| Lipoprotein synthesis and assembly | |  |  |  |  |  |
| *APOB100* | 5.26 | 3.00 | 2.71 | 2.83 | 2.77 | 0.73 |
| *MTTP* | 6.71 | 5.92 | 5.45 | 5.34 | 3.72 | 0.80 |
| Cholesterol efflux |  |  |  |  |  |  |
| *ABCA1* | 6.05 | 5.12 | 4.21 | 7.36 | 8.26 | 0.82 |
| De novo hepatic lipogenesis |  |  |  |  |  |  |
| *SREBF1* | 3.46 | 3.90 | 8.05 | 8.69 | 8.90 | 0.98 |
| Ketogenesis |  |  |  |  |  |  |
| *ACAT1* | 1.87 | 1.80 | 1.11 | 1.15 | 1.67 | 0.45 |
| *HMGCL3* | 4.37 | 4.62 | 4.23 | 5.11 | 4.44 | 0.43 |
| Cytokines |  |  |  |  |  |  |
| *IL1B* | -0.005 | 0.65 | 0.90 | 0.98 | 0.58 | 0.52 |
| Acute phase response |  |  |  |  |  |  |
| *SAA3* | 3.65 | 4.59 | 5.23 | 6.30 | 8.51 | 0.71 |
| Oxidative stress |  |  |  |  |  |  |
| *HMOX2* | 4.99 | 5.57 | 6.06 | 7.51 | 7.16 | 0.84 |
| Synthesis of antioxidants |  |  |  |  |  |  |
| *GPX3* | 7.49 | 5.67 | 5.59 | 5.01 | 4.60 | 0.94 |
| *NQO1* | 8.06 | 8.98 | 10.54 | 12.50 | 10.59 | 1.86 |
| *SOD1* | 1.52 | 0.98 | 0.18 | 1.70 | 2.10 | 0.62 |

^1^ Supplementation of 0, 6.45, 12.90, 19.35 or 25.80 g/d of choline ion as rumen-protected choline.

^2^ Cows were fed-restricted to 30% of the NE_L_ required for maintenance and pregnancy from days 6 to 14 according to NASEM [19], and hepatic tissue was collected on day 14.
