# Supplementary material for: Dose-dependent effects of rumen-protected choline on hepatic metabolism during induction of fatty liver in dry pregnant dairy cows
Source: PLoS One. 2023 Oct 5;18(10):e0290562. doi: 10.1371/journal.pone.0290562 (PMC10553221; doi:10.1371/journal.pone.0290562)
Supplement: Attachment — Submitted filename: renamed_e4c4f.docx [file pone.0290562.s005.docx]

PONE-D-23-11577

Effect of amount of rumen-protected choline on hepatic metabolism during induction of fatty liver in dairy cows

PLOS ONE

Dear Dr. Santos,

Thank you for submitting your manuscript to PLOS ONE. After careful consideration, we feel that it has merit but does not fully meet PLOS ONE’s publication criteria as it currently stands. Therefore, we invite you to submit a revised version of the manuscript that addresses the points raised during the review process.

The article is well-written and attractive in this field. Before acception, please address all the questions raised by Reviewer.

Please submit your revised manuscript by Jul 07 2023 11:59PM. If you will need more time than this to complete your revisions, please reply to this message or contact the journal office at [plosone@plos.org](mailto:plosone@plos.org). When you're ready to submit your revision, log on to <https://www.editorialmanager.com/pone/> and select the 'Submissions Needing Revision' folder to locate your manuscript file.

Please include the following items when submitting your revised manuscript:

- A rebuttal letter that responds to each point raised by the academic editor and reviewer(s). You should upload this letter as a separate file labeled 'Response to Reviewers'.
- A marked-up copy of your manuscript that highlights changes made to the original version. You should upload this as a separate file labeled 'Revised Manuscript with Track Changes'.
- An unmarked version of your revised paper without tracked changes. You should upload this as a separate file labeled 'Manuscript'.

If you would like to make changes to your financial disclosure, please include your updated statement in your cover letter. Guidelines for resubmitting your figure files are available below the reviewer comments at the end of this letter.

If applicable, we recommend that you deposit your laboratory protocols in protocols.io to enhance the reproducibility of your results. Protocols.io assigns your protocol its own identifier (DOI) so that it can be cited independently in the future. For instructions see: [https://journals.plos.org/plosone/s/submission-guidelines#loc-laboratory-protocols](https://nam10.safelinks.protection.outlook.com/?url=https%3A%2F%2Fjournals.plos.org%2Fplosone%2Fs%2Fsubmission-guidelines%23loc-laboratory-protocols&data=05%7C01%7Cjepsantos%40ufl.edu%7C168b3b21d51f44a0285308db5bb3bbbb%7C0d4da0f84a314d76ace60a62331e1b84%7C0%7C0%7C638204601281186405%7CUnknown%7CTWFpbGZsb3d8eyJWIjoiMC4wLjAwMDAiLCJQIjoiV2luMzIiLCJBTiI6Ik1haWwiLCJXVCI6Mn0%3D%7C3000%7C%7C%7C&sdata=nntQIC7nchqdglukEr%2B4qt%2B41CeZ1Ruxk%2Bn2zWGlCpc%3D&reserved=0). Additionally, PLOS ONE offers an option for publishing peer-reviewed Lab Protocol articles, which describe protocols hosted on protocols.io. Read more information on sharing protocols at [https://plos.org/protocols?utm_medium=editorial-email&utm_source=authorletters&utm_campaign=protocols](https://nam10.safelinks.protection.outlook.com/?url=https%3A%2F%2Fplos.org%2Fprotocols%3Futm_medium%3Deditorial-email%26utm_source%3Dauthorletters%26utm_campaign%3Dprotocols&data=05%7C01%7Cjepsantos%40ufl.edu%7C168b3b21d51f44a0285308db5bb3bbbb%7C0d4da0f84a314d76ace60a62331e1b84%7C0%7C0%7C638204601281186405%7CUnknown%7CTWFpbGZsb3d8eyJWIjoiMC4wLjAwMDAiLCJQIjoiV2luMzIiLCJBTiI6Ik1haWwiLCJXVCI6Mn0%3D%7C3000%7C%7C%7C&sdata=wTCLO8DycAnlzv%2BvvfQ8IxpisuEtpTUfN7508xCeC9k%3D&reserved=0).

We look forward to receiving your revised manuscript.

Kind regards,

Fei Luo
Academic Editor
PLOS ONE

**Dear Editor,**

**We thank the review process of PLOS ONE and the effort of both reviewers in providing constructive criticism to improve this manuscript. We have addressed the reviewers’ comments by providing detailed responses to their suggestions and concerns and by making the respective changes to the revised manuscript that are highlighted in yellow in the version now labeled as PONE-D-23-11577R1.**

**Below are the responses to the specific comments made by the editor/journal (responses follow AU for authors).**

**Best regards,**

**José E.P. Santos**

**University of Florida**

Journal Requirements:

When submitting your revision, we need you to address these additional requirements.

1. Please ensure that your manuscript meets PLOS ONE's style requirements, including those for file naming. The PLOS ONE style templates can be found at 
<https://journals.plos.org/plosone/s/file?id=wjVg/PLOSOne_formatting_sample_main_body.pdf> and 
<https://journals.plos.org/plosone/s/file?id=ba62/PLOSOne_formatting_sample_title_authors_affiliations.pdf>

2. We suggest you thoroughly copyedit your manuscript for language usage, spelling, and grammar. If you do not know anyone who can help you do this, you may wish to consider employing a professional scientific editing service.

**AU: The original and now the revised manuscripts have been carefully edited by the authors for proper English language usage, spelling, and grammar. No external scientific editor was used or deemed necessary. The language in the submitted article is clear, correct, and unambiguous, as indicated by both reviewers in their response.**

Whilst you may use any professional scientific editing service of your choice, PLOS has partnered with both American Journal Experts (AJE) and Editage to provide discounted services to PLOS authors. Both organizations have experience helping authors meet PLOS guidelines and can provide language editing, translation, manuscript formatting, and figure formatting to ensure your manuscript meets our submission guidelines. To take advantage of our partnership with AJE, visit the AJE website (<http://learn.aje.com/plos/>) for a 15% discount off AJE services. To take advantage of our partnership with Editage, visit the Editage website ([www.editage.com](http://www.editage.com)) and enter referral code PLOSEDIT for a 15% discount off Editage services.  If the PLOS editorial team finds any language issues in text that either AJE or Editage has edited, the service provider will re-edit the text for free.

Upon resubmission, please provide the following: 
● The name of the colleague or the details of the professional service that edited your manuscript

**AU: The original and now the revised manuscripts have been carefully edited by the authors for proper English language usage, spelling, and grammar. No external scientific editor was used or deemed necessary. The language in the submitted article is clear, correct, and unambiguous, as indicated by both reviewers in their response.**

● A copy of your manuscript showing your changes by either highlighting them or using track changes (uploaded as a *supporting information* file)

**AU: A file containing the revised version of the manuscript labeled *PONE-D-23-11577R1 highlighted* has been uploaded as “supporting information” with all changes made to the manuscript with yellow highlighting to facilitate identification of the corrections suggested by the reviewers.**

● A clean copy of the edited manuscript (uploaded as the new *manuscript* file)

**AU: A file containing a clean copy of the manuscript labeled *PONE-D-23-11577R1* has been uploaded as requested.**

3. To comply with PLOS ONE submissions requirements, in your Methods section, please provide additional information regarding the experiments involving animals and ensure you have included details on (1) methods of sacrifice, (2) methods of anesthesia and/or analgesia, and (3) efforts to alleviate suffering.

**AU: The details requested have been incorporated into the methods section of the revised manuscript.**

4. We note that the grant information you provided in the ‘Funding Information’ and ‘Financial Disclosure’ sections do not match.

When you resubmit, please ensure that you provide the correct grant numbers for the awards you received for your study in the ‘Funding Information’ section.

**AU: Information has been corrected as suggested.**

5. Thank you for stating the following in the Acknowledgments Section of your manuscript: 
"The authors thank Jorge E. Zuniga and Michael B. Poindexter (University of Florida) for their help ensuring proper daily care of cows and sample collection. The help of the staff of the University of Florida Dairy Unit (Hague, FL) is greatly acknowledged. The authors thank Barbara Barton of Balchem Animal Nutrition and Health (New Hampton, NY) for providing the rumen-protected choline product for this experiment and Dr. Peter J. Hansen (University of Florida) for allowing the use of his laboratory for the extraction of RNA. The Higher Education Commission of Pakistan partially funded the Ph.D. program of Usman Arshad. The authors have not stated any conflicts of interest."

We note that you have provided funding information that is not currently declared in your Funding Statement. However, funding information should not appear in the Acknowledgments section or other areas of your manuscript. We will only publish funding information present in the Funding Statement section of the online submission form. 
Please remove any funding-related text from the manuscript and let us know how you would like to update your Funding Statement. Currently, your Funding Statement reads as follows: 
"The Higher Education Commission of Pakistan partially funded the Ph.D. program of Usman Arshad."

Please include your amended statements within your cover letter; we will change the online submission form on your behalf.

**AU: We have removed the funding-related text from the acknowledgements’ section in the revised manuscript. The funding information has been moved to the Supporting Information file.**

6. Thank you for stating the following financial disclosure: 
"The Higher Education Commission of Pakistan partially funded the Ph.D. program of Usman Arshad."

Please state what role the funders took in the study.  If the funders had no role, please state: "The funders had no role in study design, data collection and analysis, decision to publish, or preparation of the manuscript." 
If this statement is not correct you must amend it as needed. 
Please include this amended Role of Funder statement in your cover letter; we will change the online submission form on your behalf.

**AU: Information added to the cover letter and the supporting information file. Both uploaded with the revised manuscript.**

7. We note that you have stated that you will provide repository information for your data at acceptance. Should your manuscript be accepted for publication, we will hold it until you provide the relevant accession numbers or DOIs necessary to access your data. If you wish to make changes to your Data Availability statement, please describe these changes in your cover letter and we will update your Data Availability statement to reflect the information you provide."

**AU: We will deposit the data from this article in a public repository at** [**https://datadryad.org/stash**](https://datadryad.org/stash) **once a decision on the manuscript has been made.**

8. Please include captions for your Supporting Information files at the end of your manuscript, and update any in-text citations to match accordingly. Please see our Supporting Information guidelines for more information: <http://journals.plos.org/plosone/s/supporting-information>.

**AU: We have included the funding information, declaration of competing interest, and data availability statement in the supporting information file, which is also uploaded to the online submission portal.**

9. Please review your reference list to ensure that it is complete and correct. If you have cited papers that have been retracted, please include the rationale for doing so in the manuscript text, or remove these references and replace them with relevant current references. Any changes to the reference list should be mentioned in the rebuttal letter that accompanies your revised manuscript. If you need to cite a retracted article, indicate the article’s retracted status in the References list and also include a citation and full reference for the retraction notice.

**AU: We have carefully reviewed the list of references and it is complete and correct. We did not cite any reference in the initial submission of the manuscript that is currently retracted.**

**Comments to the Authors**

1. Is the manuscript technically sound, and do the data support the conclusions?

The manuscript must describe a technically sound piece of scientific research with data that supports the conclusions. Experiments must have been conducted rigorously, with appropriate controls, replication, and sample sizes. The conclusions must be drawn appropriately based on the data presented.

Reviewer #1: Yes

Reviewer #2: Yes

2. Has the statistical analysis been performed appropriately and rigorously?

Reviewer #1: Yes

Reviewer #2: Yes

3. Have the authors made all data underlying the findings in their manuscript fully available?

The [PLOS Data policy](https://nam10.safelinks.protection.outlook.com/?url=http%3A%2F%2Fwww.plosone.org%2Fstatic%2Fpolicies.action%23sharing&data=05%7C01%7Cjepsantos%40ufl.edu%7C168b3b21d51f44a0285308db5bb3bbbb%7C0d4da0f84a314d76ace60a62331e1b84%7C0%7C0%7C638204601281186405%7CUnknown%7CTWFpbGZsb3d8eyJWIjoiMC4wLjAwMDAiLCJQIjoiV2luMzIiLCJBTiI6Ik1haWwiLCJXVCI6Mn0%3D%7C3000%7C%7C%7C&sdata=mib%2FFa7to87mPpuZXPZf2kwE9qmpyRSTNqW0akNWUMs%3D&reserved=0) requires authors to make all data underlying the findings described in their manuscript fully available without restriction, with rare exception (please refer to the Data Availability Statement in the manuscript PDF file). The data should be provided as part of the manuscript or its supporting information, or deposited to a public repository. For example, in addition to summary statistics, the data points behind means, medians and variance measures should be available. If there are restrictions on publicly sharing data—e.g. participant privacy or use of data from a third party—those must be specified.

Reviewer #1: Yes

Reviewer #2: Yes

4. Is the manuscript presented in an intelligible fashion and written in standard English?

PLOS ONE does not copyedit accepted manuscripts, so the language in submitted articles must be clear, correct, and unambiguous. Any typographical or grammatical errors should be corrected at revision, so please note any specific errors here.

Reviewer #1: Yes

Reviewer #2: Yes

**PONE-D-23-11577 Response to reviewers’ comments:**

**On behalf of all authors, I want to thank the reviewers for their suggestions to improve the manuscript and their constructive criticism of this work.**

**We have addressed every comment and suggestion made by both reviewers and the respective changes are highlighted in yellow in the revised manuscript. Responses follow the abbreviation AU below each comment.**

**Regards,**

**José E.P. Santos**

**University of Florida**

5. Review Comments to the Author

Please use the space provided to explain your answers to the questions above. You may also include additional comments for the author, including concerns about dual publication, research ethics, or publication ethics. (Please upload your review as an attachment if it exceeds 20,000 characters)

Reviewer #1: The contents of the present manuscript add on to the current knowledge of supplementing choline to dairy cows. The study is well performed and of relevance and interest not only for dairy science. However, there are some aspects as detailed below that should be addressed to improve the manuscript.
Title: admittedly, I am not a native speaker, but the term “amount” in the title sounds odd to me. To my understanding, four different doses of choline were tested, so something like “Dose-dependent effects of rumen-protected choline on hepatic metabolism during induction of fatty liver in dairy cows” would be more adequate.

**AU: We thank the reviewer for the positive comments. Amount implied quantity, but we have followed the reviewer’s suggestion and changed the title to:**

**“****Dose-dependent effects of rumen-protected choline on hepatic metabolism during induction of fatty liver in dry pregnant dairy cows”**

Abstract.
Line 20: is it important to stress Choline ION here? Why? In the later text, it was stated that choline chloride was used. Though it will likely dissociate in watery solutions, the ion was not fed as such to the animals and thus the respective statement in the abstract is not exact.

**AU: The reviewer is correct that choline was provided as a salt of choline chloride in a rumen-protected form. The rationale to use the nomenclature “choline ion” is because we want to give the reader the exact amount of choline fed. Although choline chloride is probably the most common salt used as supplement in animal feeds, choline can be presented as different salts such as choline bitartrate, commonly used as human supplement, choline hydroxide, and others. The dairy cattle literature on choline is often not specific on the exact amount of choline fed. We wanted to avoid that, although we completely agree with the reviewer that we never fed choline as an ion.**

**In the materials and methods section of the manuscript, we clearly described how choline was fed and the exact calculations used to describe the amounts of choline ion fed to cows in the experiment.**

What kind of rumen protection was used? Was the carrier feed accounted for its energy content, i.e., by administration of the carrier feed without the active compound to have the same amount of top-dressing in all four groups?

**AU:** **Choline chloride was microencapsulated with hydrogenated triacylglycerol with a proprietary technology developed by Balchem Corporation.**

[**https://balchem.com/animal-nutrition-health/technologies/choline/**](https://balchem.com/animal-nutrition-health/technologies/choline/)

**The amount of top-dress fed to each cow was not the same. All cows received exactly the same amount of the mixture of corn grain, molasses, and sodium chloride (114 g/day), but the amounts of RPC product increased from 0 to 120 g/d depending on the treatment. The product contains 28.8% choline chloride, 10.2% inert filler, and 61% hydrogenated triacylglycerol. The digestibility of the hydrogenated triacylglycerol is very low, contributing a very limited amount of net energy. For instance, cows fed 120 g/d of RPC received 73 g of hydrogenated triacylglycerol, with an estimated digestible energy content that is less than 3.1 Mcal/kg (The 3.1 Mcal of DE/kg is derived from hydrogenated palm triacylglycerol – see J. Dairy Sci. 87:1446–1454, which is more digestible that hydrogenated soybean oil, the latter resulting in mostly triacylglycerol rich in stearic acid). In any case, if the digestible energy is 3.1 Mcal/kg, then the cows fed 120 would receive an extra 0.22 Mcal of digestible energy per day, or approximately 0.19 Mcal of net energy for lactation per day.**

**We have added a sentence to clarify that the amounts of top dress were not the same for all treatments. Although the amount of energy offered daily differed because of the added hydrogenated triacylglycerol, we believe that the difference in energy intake is negligible. However, if the reviewer believes we should describe those calculations in the text and indicate to the reader the additional net energy supplied with the added RPC product for each treatment, we would be glad to do so. The extra net energy supplied relative to treatment with 0 RPC were 0.048, 0.095, 0.143, and 0.190 Mcal of net energy for lactation per day.**

Line 22: Were primiparous or pluriparous cows enrolled, or both? Information on this is provided later, but should also be included in the abstract.

**AU: We enrolled 35 primiparous and 41 multiparous cows in this experiment. This information has been added to the abstract and presented in the text of the materials and methods of the revised manuscript.**

Line 23: the digits in the dose information should be limited to three, more is not more precise (e.g., 12.9 instead of 12.90). Use numbers as you did in bold print in Lines 110 – 112.

**AU: We understand that, but we never measured the amount of choline ion to be fed daily. We measured the amount of the RPC product fed. If one does the math, if we use a single digit to make the language simpler, then feeding 30 g of RPC, which provided 6.45 g of choline ion, to become 6.5 g of choline ion, then when we fed 120 g of RPC, the dose of choline ion would be 26.0 g, which is not correct. The correct amount was 25.80 g of choline ion. We understand this is a small difference, but we would rather describe the exact amount that was fed. We labeled the treatment with a single digit to be easier for the reader to follow the text, but we prefer to keep the amounts closer to what was fed. We hope the reviewer understands our position.**

Line 24; It is not quite clear to what the day mentioned refers, is it day of lactation? In any case, please indicate accordingly. How were the cows fed before calving?

**AU: We used dry cows for the experiment. Cows were enrolled at a mean of 211 days of gestation. Days 1 to 14 refer to days in the experiment. All cows enrolled in the experiment had calved at least once, but they were recently dried off before the experiment started. We did use any cows that was lactating in the experiment. This is clearly stated throughout the materials and methods.**

**Days 1 to 5 of the experiment refer to the ad libitum feeding period and days 6 to 14 of the experiment refer to the feed restriction period.**

**We included “dry pregnant” in the title of the manuscript to avoid any confusion.**

Line 27: “mRNA expression” cannot be directly measured, only mRNA abundance. This should be stated as such and also some specification of the target genes should be provided.

**AU: We changed the word expression to abundance in the abstract and throughout the manuscript. We had 93 transcripts representing several pathways associated with glucose and lipid metabolism. PLoS One only allows 300 words in the abstract and adding information on all these genes would exceed the words limit. We have explained details of target genes and their associated pathways in the materials and methods section of the manuscript.**

Line 32: what means “as is basis”?

**AU: This refers to the hepatic tissue on wet-basis. We changed the term as is to wet-basis in the abstract of the revised manuscript.**

Line 33: change expression to abundance and do so throughout the entire manuscript.

**AU: Changed as suggested.**

Line 42: last week or weeks??

**AU: We would like to keep it as last week.**

**If one looks at plots of dry matter intake for individual cows prepartum (not means of groups of cows), the drop in intake really happens in the last few days of gestation. This is particularly noticeable in diets with high forage and forage NDF content. Because of that, the calculated net energy balance become negative mostly in the last week of gestation, but not before that.**

Line 53 – 55: Is supplementing choline as inferred in these lines always physiologically necessary for ruminants, or only for high yielding dairy cows?

**AU: In our experiments with dry cows (not lactating), supplementing RPC consistently reduces triacylglycerol concentration in the liver, as it does for almost every other species studied. The fact these cows are dry implies that lactation is not influencing the response to choline. We do not believe the mechanisms of action of choline on the cow is dependent on her genetic merit.**

**To address the question of whether production responses to supplementing RPC depend on the level of production of cows, we went back to our meta-analysis of 2020 (Arshad et al. 2020 J. Dairy Sci. 103:282–300) and re-analyzed the data according to the observed production of cows in the different experiments. See the Forest plot below. The overall response was an increment of 1.87 kg with supplemental RPC.**

**When we segregated the data according to the median ECM yield of all experiments, 36 kg/d, as above or below, the mixed-model for the weighted mean difference of ECM yield was an increment of 1.64 kg/d for cows producing > 36 kg/d and 1.49 kg/day for experiments in which cows produced less than 36 kg of ECM/day.**

Line 71: see comment to the title (amounts)

**AU: We have followed the reviewer’s suggestion and changed the title to:**

**“Dose-dependent effects of rumen-protected choline on hepatic metabolism during induction of fatty liver in dry pregnant dairy cows”**

Line 75: delete “of” after dispose

**AU: It is correct as is. To dispose is a transitive verb that requires the preposition of after it. “to dispose of”....**

Line 97 – 99. The diet of the pregnant dry cows is not mentioned; assumingly they were not fed ad libitum and if the diet was changed only with calving, this would affect the adaptive capability of the animals. At least the energy content of the dry cows` diet should be provided.

**AU: We would like to clarify that the experiment was performed with only dry cows. Cows in the experiment had been dried off and they were 211 days of gestation when the experiment started.**

**Details are presented in the text of the manuscript and the information on the ingredients and nutrient contents of the diet in depicted in Supplemental Table 1.**

Lines 103 – 104: the days of the experiment seem to equal with days of lactation. However, this would imply that the choline supplementation also started only at day 1 immediately after calving. Please make it very clear what day 1 of the experiment is (see also Lines 117 – 118).

**AU: The experiment was performed with only dry pregnant Holstein cows (no cow in the experiment was lactating). Days 1 to 5 refer to the ad libitum period and days 6 to 14 refer to the feed restriction period. We believe this is clear now.**

Line 146 vs. Line 149: the days of sampling are not consistent.

**AU: Thank you for catching this. We collected hepatic tissues on days 5 and 14 of the experiment. We corrected this typo error in the revised manuscript.**

Line 150: 25 mg were (not was)

**AU: Corrected.**

Lines 158 – 161: what about RNA quality, e.g., RIN numbers?

**AU: AU: We did not analyze the RNA integrity number in every sample. We analyzed the RIN using an Agilent bioanalyzer in a random set of 20 samples (4/treatment) at the University of Florida core lab before performing the PCR reactions and they all resulted in RIN equal to or larger than 6.8 (RIN of 6.8 to 9.5).**

**Because we used RNAse free vials, used RNA later, and froze samples in liquid N_2_ immediately upon collection and use aseptic measures, and the set of 20 samples resulted in high integrity RNA, we decided to not run RIN in every sample. Maybe we overlooked that, but we had no indication of issues with RNA quality.**

**For the purity of RNA, samples on days 5 and 14 had a mean (± standard deviation) 260:280 nm ratio of 2.25 ± 0.22 (range 1.98 to 2.85), and 2.16 ± 0.11 (range 1.99 to 2.42), respectively. The values of 260:280 ratio is acceptable for the purity of RNA and indicates that RNA is not contaminated with proteins, phenols or other contaminants that absorb strongly at or near 280 nm. We added this information to the materials and methods section of the revised manuscript.**

Line 354: change to phospholipid (singular form)

**AU: Changed.**

Lines 353 – 357: Provide adequate reference (-s) for the statements about the Kennedy pathway, not every potential reader would know it.

**AU: Reference added as suggested.**

Line 373: change “expression” to “mRNA abundance” (see also Line 376, and several cases in the following sections, and also the figure captions)

**AU: Expression has been replaced with abundance throughout the manuscript as suggested.**

Line 381: could you relate the micro molar concentrations from this in vitro study to the blood (and maybe liver) concentrations in the cows from the present study? Would they be in the same order of magnitude?

**AU: The concentrations of choline chloride (74.6% choline ion) used by Chandler and White 2017 in their cell culture were 0, 61, 128, 2028, and 4528 micromoles/L, thus resulting ins approximately 0, 45.5, 95.5, 1,512, and 3,378 micromoles/L of choline ion.**

**In a live animal, choline is present in numerous forms (e.g., free choline ion, phosphocholine, numerous lysophosphatidylcholines, numerous phosphatidylcholines, sphingomyelin, etc.)**

**Michael DeVeth in his paper in 2016 (J. Dairy Sci. 99:9732–9744) measured choline in arterial blood of dairy cows producing 31 kg of milk/day and consuming 21 kg of DM per day (those cows were supplemented with different amounts of choline). The main forms of choline detected were numerous phosphatidylcholines. The concentrations they found ranged from 7,300 to 9,800 microles/L. Assuming a mean molecular weight of phosphatidylcholine of 776 grams/mol, then choline ion would represent ~13.4%. Thus, the concentrations of choline ion (as phosphatidylcholines) in arterial blood of cows in DeVeth’s experiment would range from 978 to 1,313 micromoles/L. The fourth level treatment by Chandler and White (2017), 1,512 micromoles/L of choline ion as choline chloride, would be in the upper content of arterial choline, whereas all others would represent very low concentrations or very high concentration relative to the findings of DeVeth.**

**We are not aware of concentration of choline ions in hepatic tissue to make a parallel with the data from Chandler and White.**

**Let us know if you want us to add any of that information in the discussion.**

Lines 424 – 426: would attenuating the degree of inflammatory response by RPC be always beneficial, could you speculate on reduced immune response in case of infections?

**AU: That is an excellent point and not an easy one to objectively answer.**

**In general, dietary interventions (not pharmacological – administering anti-inflammatory drugs such as NSAIDs or steroids can have a multitude of effects, some positive, some very negative) that attenuate the inflammatory and innate immune responses, they do not seem to increase the risk of new infections. For instance, extensive data on n3 fatty acids, particularly alpha-linolenic, eicosapentaenoic, and docosahexaenoic acids show that they reduce activation of inflammatory cascade, and feeding experiments do not show an increase in risk of diseases when those fatty acids are supplemented to the diet. In many cases, the risk of disease is reduced, although the diseases evaluated are not always of infectious nature.**

**In the meta-analysis of choline (Arshad et al., 2020), we were able to identify incidence of diseases in half (11 papers reported health events) of the published papers that fitted the inclusion criteria. Supplementing RPC tended to reduce the risk of retained placenta and that of mastitis in dairy cows. Obviously, we cannot infer that the benefits to health were mediated by possible anti-inflammatory effects of phosphatidylcholines. Nevertheless, it provides some indication that feeding choline and its associated effects do not increase the risk of diseases in cows.**

Line 441: what are “parous cows”?

**AU: Cows that have calved at least once previously. We changed to multiparous.**

Line 469: in this case choline ion?? Hepatic is rather hepatocytic?

**AU: The authors (Chandler and White, 2017 PLoS One 14: e0217160) treated primary monolayer hepatocytes cell line with choline chloride. We would like to keep the word “hepatic” as is.**

Conclusions: coming back to the title, could you conclude which dosage is optimal in your feed restriction challenge, and may be without such challenge?

**AU: Our interpretation, based on hepatic composition, is that we were unable to detect an optimal amount of choline ion to be fed to cows within the range studied here. The response to choline ion for triacylglycerol was mostly linear. Some of the mRNA responses were not necessarily linear with dose of choline ion fed, but we do not think we can identify a particular amount that was optimum for cows. If one looks at the mRNA expression, many response were quadratic, which would infer that the mean between 12.9 and 19.35 g/d was optimum.**

**In another experiment, we used a similar model and fed 110 cows 0, 12.9, or 25.8 g/d of choline ion and most responses were linear (Arshad et al. 2023 J. Dairy Sci. 106: accepted). In the 2020 meta-analysis (Arshad et al. 2020 J. Dairy Sci. 103:282–300), increasing the intake of choline ion up to 25.2 g/d resulted in linear increases in yields of milk, ECM, etc.**

**At this point, we do not think the literature can support a particular amount of choline ion that is ideal for cows at different physiological states.**

References: ref. No 32 is problematic since only under review, so you can`t use it as such. Any progress with that one?

**AU: The references listed at number 32 and 33 have been accepted for publication in JDS. We have added the DOI number of reference 32 and would add the DOI number of reference 33 once available.**

Figure 4: which software was used to generate the schematic? In case of Biorender it would need to be referenced. Moreover, in this visualization, it would be helpful to have the full gene names without having to jump to the supplementary information.

**AU: Yes, we used BioRender software and the reference with the link to the page has been added as suggested. We included the gene names with the respective gene abbreviation in the footnote of the figure.**

Reviewer #2: The article is a very nice contribution to the study of dosage of RPC to cows subjected to fatty liver and to the molecular action of choline.

**AU: We thank the reviewer for the positive comments. The comments/suggestions of the reviewer have been addressed and changes have been incorporated and highlighted in yellow in the revised manuscript.**

Abstract:
L21: “…subjected to a fatty liver induction protocol.”

**AU: Changed as suggested.**

L21: Define: Pregnant, non-lactating Holstein cows with at least one lactation.

**AU: Agreed. The parity group of the cows has been clarified in the revised manuscript.**

L23-24: Define the duration of RPC supplementation (14 d ? 5 ad lib + 9 feed restricted) and how it was supplemented to guarantee the intake of the daily dosages (Top-dress).

**AU: Yes, the cows were supplemented with RPC for 14 days and the doses were fed as top-dressed. The information on duration of RPC and top-dressing has been included in the revised manuscript.**

**During the ad libitum period, first 5 days, we cannot guarantee that cows consumed 100% of the top dress because we fed them for ad libitum intake and some top dress could have gone to the bottom of the feeder and the cow would not consume everything. However, during the feed restriction period, all 76 cows clean the bunk every day. There was nothing left (we used large plastic bucket feeders).**

Material and Methods:
L111: Describe product ReaShure: company, country.

**AU: The information is in line 107 of the revised manuscript.**

L113-115: Make clear that the treatments were fed only for 14 days (5 + 9).

**AU: The information on the duration of RPC has been added to the revised manuscript.**

L124: Is it NASEM 2021 predicted supply? I would report the predicted metabolizable AA efficiency, if this model was used to report Met supply.

**AU: Yes, the values we are reporting are the predicted supply of metabolizable protein and metabolizable methionine based on the composition of the diet and adjusted for the observed dry matter intake using the NASEM 2021 software.**

**NASEM uses 0.69 for efficiency of metabolizable protein use for all tissues except for incorporation into pregnant uterus and for excretion as urinary N. For pregnancy, the efficiency of MP use by NASEM 2021 is 0.33 and for urinary N excretion is 1. Although efficiency on use of different amino acids by the mammary gland differ, NASEM does not discriminate that for use by other tissues, which would be the case for nonlactating cows.**

**We added the efficiency of use of metabolizable amino acids reported by NASEM (0.69, 0.33, and 1) in the text.**

L125: Avoid paragraph with only one sentence.

**AU: Changed as suggested.**

L135: I would report the actual DMI ± SD during the period of feed restriction (kg/d and % of BW).

**AU: The exact mean DMI and SD were added in kg/d and as % of the body weight of the cows as requested.**

L137: I would report metabolizable MET predicted efficiency in addition to the prediction of g/d.

**AU: See previous comment. NASEM does not discriminate efficiency of use of each individual amino acid in nonlactating animals.**

Results:
L261: P < 0.01. No need to use 3 decimals (0.001).

**AU: We truncated the P-values to 3 decimal cases throughout the manuscript.**

L285: P < 0.01. In place of P = 0.007.

**AU: We would rather provide the exact P-value up to the 3 decimal cases.**

L289: P < 0.01.

**AU: See comment above.**

L334: Avoid paragraphs with only one sentence.

**AU: Changed as suggested.**

Discussion:
Is it possible to provide some discussion on why RPC increased (P = 0.06 for linear) triacylglycerol (ug/ug DNA) during ad libitum feeding (L249-250) and reduced it during feed restriction (L284-285)? There was a cubic reduction during feed restriction (P = 0.05). Does it have a biological meaning?

**AU: We did not necessarily anticipate the linear increase in triacylglycerol with increased dose of choline ion (from 6.45 to 25.8 g/d) during the ad libitum period. We expected that there would be no evidence for a treatment effect. Nevertheless, the orthogonal polynomial for the linear contrast is measuring the effect of the mean between 6.45 and 12.9 vs. the mean between 19.35 and 25.8 g/d. For the ad libitum period, those means were 2.00 and 2.33 ug/ug DNA. Although the effect is there, the magnitude of difference (0.33 ug/ug DNA) is very small and the content of triacylglycerol in the tissue also was very small (~ 0.70% of the tissue wet-basis), typical of well-fed cows. We do not have a clear explanation for that, other than the fact that this finding probably has limited relevance.**

**Now, when cows are at risk of developing hepatic lipidosis, our assumption is that molecules that facilitate lipid transport and export by the liver would be more beneficial. When we imposed feed restriction, cows fed 0 g/d of choline ion experienced a 9-fold increase in hepatic triacylglycerol content. Benefits from lipotropic molecules are likely to be enhance under those circumstance. Also, when cows were subjected to feed restriction, to mimic a period or negative nutrient balance such as in cows in early lactation, the supply of nutrients that might be used to synthesis phospholipids are probably limiting under those circumstances (e.g., endogenous synthesis/recycling of choline molecules such as methylation of phosphatidylethanolamines to produce phosphatidylcholines, etc.).**

**To address your last point, whether the mixed effects (linear increase during ad libitum and a linear decrease during feed restriction) have a biological meaning, we believe it does. The obvious answer is that under feed restriction, the needs for choline increase and the expected effects are those typical of a lipotropic molecule with benefits to reduce the risk of steatosis; however, during periods of no risk of steatosis, the benefits of supplementing the diet with RPC is nonexistent relative to reducing hepatic triacylglycerol. It might have other effects in the body, but it is no longer important to reduce hepatic triacylglycerol as the content of that is very small in the tissues.**

**We did not provide discussion on the increase in hepatic triacylglycerol during the ad libitum period because we felt the magnitude was very small. If the reviewer believes we should add discussion on that, we will incorporate some of what we mentioned in the response herein. Let us know.**

6. PLOS authors have the option to publish the peer review history of their article ([what does this mean?](https://nam10.safelinks.protection.outlook.com/?url=https%3A%2F%2Fjournals.plos.org%2Fplosone%2Fs%2Feditorial-and-peer-review-process%23loc-peer-review-history&data=05%7C01%7Cjepsantos%40ufl.edu%7C168b3b21d51f44a0285308db5bb3bbbb%7C0d4da0f84a314d76ace60a62331e1b84%7C0%7C0%7C638204601281186405%7CUnknown%7CTWFpbGZsb3d8eyJWIjoiMC4wLjAwMDAiLCJQIjoiV2luMzIiLCJBTiI6Ik1haWwiLCJXVCI6Mn0%3D%7C3000%7C%7C%7C&sdata=J6nsXDoc4FuQ%2ByWnJh3qbFOVrNvd4%2Fq%2FjnQExhwpONU%3D&reserved=0)). If published, this will include your full peer review and any attached files.


If you choose “no”, your identity will remain anonymous, but your review may still be made public.


**Do you want your identity to be public for this peer review?** For information about this choice, including consent withdrawal, please see our [Privacy Policy](https://nam10.safelinks.protection.outlook.com/?url=https%3A%2F%2Fwww.plos.org%2Fprivacy-policy&data=05%7C01%7Cjepsantos%40ufl.edu%7C168b3b21d51f44a0285308db5bb3bbbb%7C0d4da0f84a314d76ace60a62331e1b84%7C0%7C0%7C638204601281186405%7CUnknown%7CTWFpbGZsb3d8eyJWIjoiMC4wLjAwMDAiLCJQIjoiV2luMzIiLCJBTiI6Ik1haWwiLCJXVCI6Mn0%3D%7C3000%7C%7C%7C&sdata=wkdVcagaNPyaDFDzciYPJY0%2Bczw5INmxOOvV%2F%2F3IyMo%3D&reserved=0).

Reviewer #1: No

Reviewer #2: No

[NOTE: If reviewer comments were submitted as an attachment file, they will be attached to this email and accessible via the submission site. Please log into your account, locate the manuscript record, and check for the action link "View Attachments". If this link does not appear, there are no attachment files.]

While revising your submission, please upload your figure files to the Preflight Analysis and Conversion Engine (PACE) digital diagnostic tool, [https://pacev2.apexcovantage.com/](https://nam10.safelinks.protection.outlook.com/?url=https%3A%2F%2Fpacev2.apexcovantage.com%2F&data=05%7C01%7Cjepsantos%40ufl.edu%7C168b3b21d51f44a0285308db5bb3bbbb%7C0d4da0f84a314d76ace60a62331e1b84%7C0%7C0%7C638204601281342641%7CUnknown%7CTWFpbGZsb3d8eyJWIjoiMC4wLjAwMDAiLCJQIjoiV2luMzIiLCJBTiI6Ik1haWwiLCJXVCI6Mn0%3D%7C3000%7C%7C%7C&sdata=lIlwiP7xbMEWHvP5VGk5oG0Nd4GfEjCHt4l5hBErsi4%3D&reserved=0). PACE helps ensure that figures meet PLOS requirements. To use PACE, you must first register as a user. Registration is free. Then, login and navigate to the UPLOAD tab, where you will find detailed instructions on how to use the tool. If you encounter any issues or have any questions when using PACE, please email PLOS at [figures@plos.org](mailto:figures@plos.org). Please note that Supporting Information files do not need this step.
